# A Novel Collaborative Filtering Model-Based Method for Identifying Essential Proteins

**DOI:** 10.3389/fgene.2021.763153

**Published:** 2021-10-21

**Authors:** Xianyou Zhu, Xin He, Linai Kuang, Zhiping Chen, Camara Lancine

**Affiliations:** ^1^ College of Computer Science and Technology, Hengyang Normal University, Hengyang, China; ^2^ Hunan Provincial Key Laboratory of Intelligent Information Processing and Application, Hengyang, China; ^3^ College of Computer, Xiangtan University, Xiangtan, China; ^4^ College of Computer Engineering and Applied Mathematics, Changsha University, Changsha, China; ^5^ The Social Sciences and Management University of Bamako, Bamako, Mali

**Keywords:** essential proteins, collaborative filtering model, PDI network, data integration, prediction model

## Abstract

Considering that traditional biological experiments are expensive and time consuming, it is important to develop effective computational models to infer potential essential proteins. In this manuscript, a novel collaborative filtering model-based method called CFMM was proposed, in which, an updated protein–domain interaction (PDI) network was constructed first by applying collaborative filtering algorithm on the original PDI network, and then, through integrating topological features of PDI networks with biological features of proteins, a calculative method was designed to infer potential essential proteins based on an improved PageRank algorithm. The novelties of CFMM lie in construction of an updated PDI network, application of the commodity-customer-based collaborative filtering algorithm, and introduction of the calculation method based on an improved PageRank algorithm, which ensured that CFMM can be applied to predict essential proteins without relying entirely on known protein–domain associations. Simulation results showed that CFMM can achieve reliable prediction accuracies of 92.16, 83.14, 71.37, 63.87, 55.84, and 52.43% in the top 1, 5, 10, 15, 20, and 25% predicted candidate key proteins based on the DIP database, which are remarkably higher than 14 competitive state-of-the-art predictive models as a whole, and in addition, CFMM can achieve satisfactory predictive performances based on different databases with various evaluation measurements, which further indicated that CFMM may be a useful tool for the identification of essential proteins in the future.

## Introduction

Researches show that essential proteins are not only important for survival of organisms but also play critical roles in the development of life processes. Hence, it is of practical significance to identify potential essential proteins (Meng et al., 2021). With the development of biotechnologies, some essential proteins have been identified successively by traditional biological experiments such as single gene knockouts (Giaever et al., 2002), RNA interference (Cullen and Arndt, 2005), and so on. However, since these traditional biological experiments are quite time consuming and expensive, it has become a hot topic to predict essential proteins by developing computational models (Wang et al., 2013). Up to now, a large number of computational models have been developed to detect essential proteins based on protein–protein interaction (PPI) networks, which can be roughly classified into two major categories. Among them, the first category of models focuses on adopting only topological features of PPI networks to predict essential proteins. For instance, based on the rule of centrality–lethality proposed (Jeong et al., 2001), a series of models, such as DC (Degree Centrality) (Hahn and Kern, 2005), SC (Subgraph Centrality) (Estrada and Rodríguez-Velázquez, 2005), BC (Betweenness Centrality) (Joy et al., 2005), EC (Eigenvector Centrality) (Bonacich, 1987), IC (Information Centrality) (Stephenson and Zelen, 1989), CC (Closeness Centrality) (Wuchty and Stadler, 2003), and NC (Neighbor Centrality) (J. Wang et al., 2012), have been designed in succession for inferring essential proteins based on topological features of PPI networks. Except for these models, Li et al., 2011) proposed a novel model called LAC to predict potential essential proteins based on neighborhoods of protein nodes in PPI networks. B. Xu et al. (2019) developed a model to detect essential proteins by applying random walks on PPI networks. Wang et al. (2011) presented a model called SoECC based on edge clustering coefficients to infer essential proteins. Qin et al. (2016) designed a method called LBCC based on characteristics of PPI networks to predict essential proteins. However, due to the incompleteness of PPI networks, all these first category of models cannot achieve satisfactory prediction accuracies of potential essential proteins.

In order to overcome the incompleteness of PPI networks, in recent years, another category of models have been proposed by integrating topological features of PPI networks and some biological information of proteins to infer essential proteins. For example, Chen et al. (2017) developed a computational model to infer essential proteins by combining PPI networks with gene ontology and KEGG pathway. Zhang X. et al. (2018) presented a prediction model by combing gene expression data with PPI networks to predict essential proteins. W. Peng et al. (2015a) proposed a prediction model called UDoNC by integrating protein domains with PPI networks to infer essential proteins. Jiang et al. (2015) developed a method called IEW to detect key essentials by combining domain interactions and topological features of PPI networks. Zhao et al. (2019) put forward a prediction model called RWHN to infer key proteins by integrating PPI networks with protein domains and some other biological information. Lei et al. (2018) put forward a prediction model named RSG by integrating subcellular localization and GO data of proteins with PPI networks to infer key proteins. Y. Fan et al. (2016) proposed a novel prediction model by adopting Pearson correlation coefficients and subcellular localization to update the PPI network Qin et al. (2017) put forward a method for recognizing essential proteins based on the topological information of PPI networks and orthologous information of proteins. Peng et al. (2012) proposed an advanced iterative algorithm named ION for identifying key proteins based on the topological information of PPI networks and homologous information of proteins. Li et al. (2012) put forward a novel prediction method called Pec through integrating the PPI network with the gene expression of proteins to improve the accuracy of the prediction model. Zhang et al. (2013) presented a novel calculation model named CoEWC by combining PPI networks with the gene expression profiles of proteins to recognize potential key proteins. Liu et al. (2020) proposed a novel prediction model named DEP-MSB by integrating biological features of proteins and topological features of PPI networks. Zhao et al. (2014) put forward an advanced iterative algorithm named POEM for detecting key proteins through combining gene expression data of proteins and topological properties of PPI networks to infer key proteins. Fang et al. (2018) proposed a novel feature selection model named ESFPA by adopting improved swarm intelligence to identify key proteins. Liu et al. (2018) developed an advanced model named EPPSO to recognize key proteins through utilizing improved particle swarm optimization. Zhang W. et al. (2018) presented a computational model called TEGS to recognize key proteins by combining biological information of proteins and topological features of PPI networks. S. Li et al. (2020) developed a novel prediction model called CVIM by combining PPI networks and orthologous information of proteins for inferring essential proteins. Z. Chen et al. (2020) presented a novel strategy named NPRI by combining various biological data of proteins and the topological features of PPI networks to infer key proteins. Although the second category of methods can greatly improve the predictive accuracy of potential essential proteins, it remains to be a challenging work to scientifically integrate topological features of PPI networks and biological features of proteins to effectively improve the accuracy of essential protein prediction.

Inspired by the above methods, in this paper, a novel Collaborative Filtering Model-based Method (CFMM) was proposed to predict potential essential proteins, in which, an original protein–domain interaction (PDI) network was constructed first, and then, considering that the number of known interactions between domains and proteins was quite limited, an updated PDI network was built by applying the collaborative filtering algorithm on the original PDI network. Next, based on the updated PDI network, some key topological features and biological features of proteins were extracted, which would be further integrated together to infer potential essential proteins based on an improved PageRank algorithm. Finally, in order to estimate the performance of CFMM, it was compared with 14 competitive prediction models such as DC (Hahn and Kern, 2005), SC (Estrada and Rodríguez-Velázquez, 2005), BC (Joy et al., 2005), EC (Bonacich, 1987), IC (Stephenson and Zelen, 1989), CC (Wuchty and Stadler, 2003), NC (J. Wang et al., 2012), ION (Peng et al., 2012), Pec (Li et al., 2012), CoEWC (Zhang et al., 2013), POEM ((Zhao et al., 2014), TEGS (Zhang W. et al., 2018), CVIM (S. Li et al., 2020), and NPRI (Z. Chen et al., 2020) based on three kinds of well-known public databases. And as a result, CFMM can achieve better prediction accuracies than all these competing methods.

## Materials

In this section, in order to construct the original PPI network, we first downloaded known PPI data from the DIP database (Xenarios et al., 2002), the Krogan database (Krogan et al., 2006) and the Gavin database (Gavin et al., 2006) separately. After removing self-interactions and repeated interactions, we finally obtained 1,167 essential proteins, 3,926 nonessential proteins, and 24,743 known interactions between 5,093 proteins from the DIP database, 14,317 known interactions between 3,672 proteins from the Krogan database, and 7,669 known interactions between 1855 proteins from the Gavin database, respectively. Moreover, we downloaded the dataset of 1,107 different domains from the Pfam database (Bateman et al., 2004). The subcellular localization data from the COMPARTMENTS databases (X. Peng et al., 2015b), (Binder et al., 2014), which consists of 4,865 proteins involved in 11 kinds of subcellular localizations, including the cytoskeleton, mitochondrion, nucleus, peroxisome, plasma, extracellular, endosome, vacuole, endoplasmic, cytosol, and Golgi. Additionally, The gene expression data were provided by Tu et al. (2005), which include 6,777 gene expressions products and 36 samples. The dataset of orthologous information of proteins are from the InParanoid database (Östlund et al., 2010), which includes a collection of pairwise comparisons between 100 whole genomes. Finally, in order to verify the accuracy of CFMM, we further downloaded a set of 1,293 essential genes from four diverse databases such as MIPS (Mewes et al., 2004), DEG (Zhang and Lin, 2009), SGD (Cherry et al., 1998), and SGDP (*Saccharomyces* Genome Deletion Project, 2012) separately. The detailed information of datasets downloaded from the DIP, Krogan, and Gavin databases are shown in the following Table 1.

**TABLE 1:** Detailed information of datasets downloaded from the DIP, Krogan, and Gavin databases.

database	Proteins	Interactions	Essential proteins	Gene expression
DIP	5,093	24,743	1,167	4,981
Krogan	3,672	14,317	929	3,610
Gavin	1,855	7,669	714	1,827

## 3 Method

**FIGURE 1:**
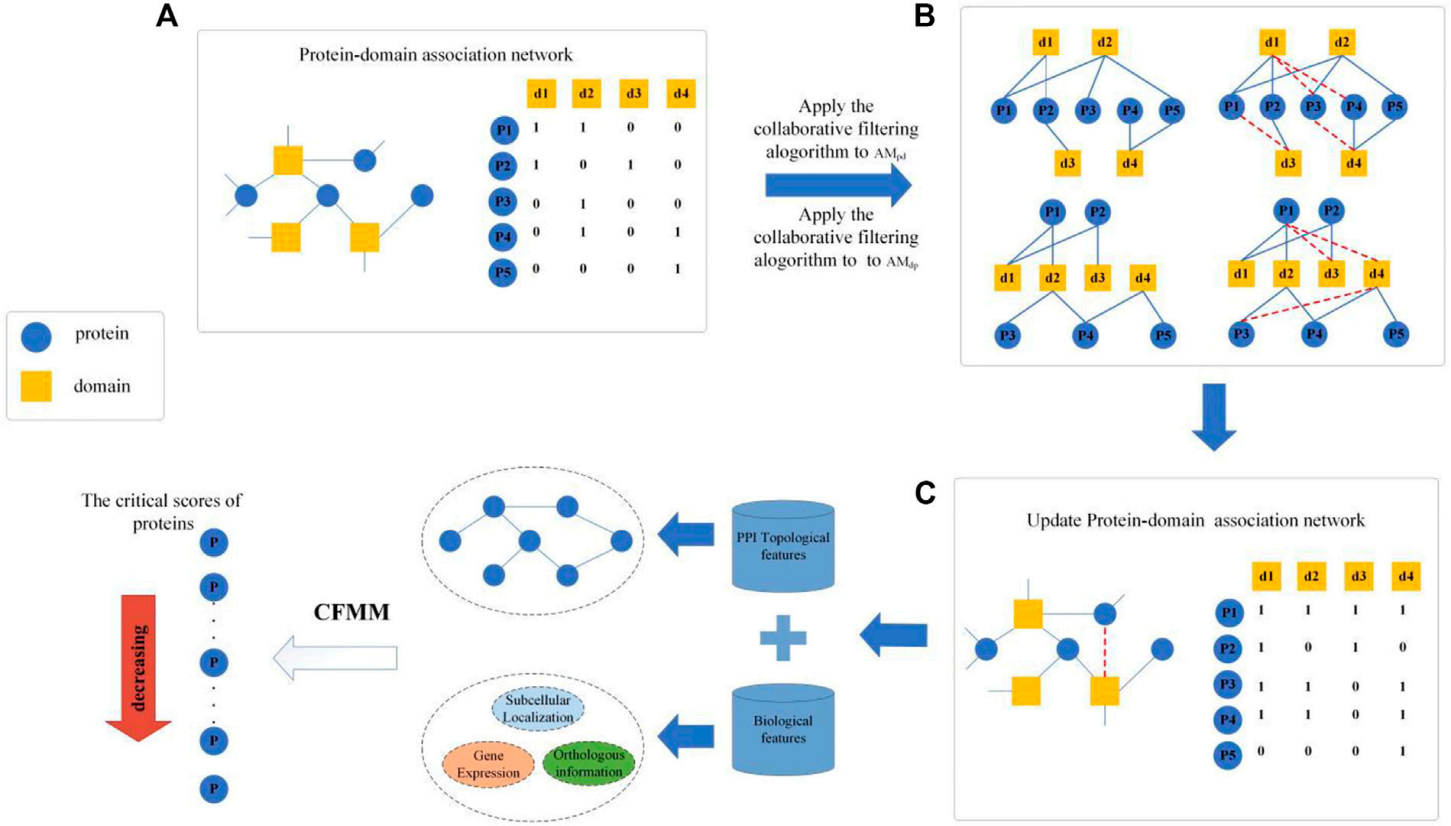
Flowchart of collaborative filtering model-based method (CFMM).

As illustrated in Figure 1, CFMM consists of the following three major steps:

**Step 1:** First, an original PDI network will be constructed based on known protein–domain interactions downloaded from given public databases, and then, a recommendation matrix will be obtained by applying the collaborative filtering algorithm on the original PDI network.

**Step 2:** Next, based on known PPI data and biological information of proteins downloaded from public databases, key topological features and biological features of proteins will be extracted separately, and then, an improved entropy weight method will be applied to effectively integrate all these features.

**Step 3:** Finally, based on a newly designed distribution rate matrix, an iterative algorithm will be proposed to infer potential essential proteins based on an improved PageRank algorithm.

### Construction of Protein–Domain Interaction

Based on known protein–domain interactions downloaded above, we can first construct an original network PDI as follows: for any given protein node $p_{i}\;$ and domain node $\;d_{j}$ , if and only if there is a known interaction between them, there is an edge between *p*_*i*_ and $\;d_{j}$ in PDI. Then we can further obtain an adjacency matrix $\;AM_{pd}$ as follows: for any given protein *p*_*i*_ and domain $\;d_{j}$ , if and only if there is a known interaction between $p_{i}$ and $\;d_{j}$ , there is $AM_{pd}\left(p_{i},d_{j}\right)$ *=* 1; otherwise, there is $AM_{pd}\left(p_{i},d_{j}\right)\;=0$ . Due to limited known PDI, obviously, $AM_{pd}$ is a sparse matrix. Hence, in order to improve the density of $AM_{pd}$ , we will apply the collaborative filtering algorithm on $AM_{pd}$ according to the following steps:

**Step 1:** Applying the protein-based collaborative filtering algorithm on PDI as follows:

First, based on $AM_{pd}$ and PDI, we will construct a novel co-occurrence matrix $CM_{PP}$ as follows: for any two given proteins $p_{i}$ and $p_{j}$ , there is $\;CM_{PP}\left(p_{i},p_{j}\right)\;=\;1$ , if and only if there is at least one common domain node existing between them; otherwise, there is $\;CM_{PP}\left(p_{i},p_{j}\right)=0$ . Hence, a similarity matrix SMPP between protein and protein can be calculated after normalizing $CM_{PP}$ as follows:

$$\mathrm{SMPP}\left(p_{i},p_{j}\right)=\{\begin{array}{cc} \frac{\left|N\left(p_{i}\right)\cap N\left(p_{j}\right)\right|}{\sqrt{\left|N\left(p_{i}\right)\right|\times \left|N\left(p_{j}\right)\right|}}: & if\;i\neq j\\ 0: & \mathrm{Otherwise} \end{array}$$ (1)

Here, $\left|N\left(p_{i}\right)\right|$ denotes the number of known domains associated to $p_{i}$ in PDI; in other words, it denotes the sum of elements equaling to one in the $i^{th}$ row of $AM_{pd}$ . $\left|N\left(p_{i}\right)\cap N\left(p_{j}\right)\right|$ represents the number of known domains related to both $p_{i}$ and $p_{j}$ simultaneously.

Based on matrices $\;AM_{pd}$ and SMPP , we can further obtain a novel recommendation matrix RMPD as follows:

$$RMPD=SMPP\times AM_{pd}$$ (2)

Next, for any given protein node $p_{i}$ and domain node $d_{j}\;$ in PDI, if the interaction between $p_{i}$ and $d_{j}$ is associated already, then for a protein node $p_{k}$ other than $p_{i}$ , it is no doubt that the higher the similarity between $p_{k}$ and $p_{i}$ , the more possibility that there may exist a potential association between $\;p_{k}$ and $d_{j}$ . Thereafter, we can define the recommendation standard between protein $p_{k}$ and $d_{j}\;$ based on the similarities between proteins as follows:

$$Std_{pk\;dj}=\frac{1}{N}\times \overset{N}{\underset{i=1}{\sum }}RMPD\left(p_{i},d_{j}\right)$$ (3)

Here, N denotes the number of proteins in PDI. Based on the above Eq. 3, for any given domain node $d_{j}$ , if there is a protein node $\;p_{k}\;$ satisfying $RMPD\left(\;p_{k},d_{j}\right)>Std_{\;p_{k}\;d_{j}}$ , then we will further recommend the protein $p_{k}$ to the domain $d_{j}$ . Thereafter, we will add a new association edge between $p_{k}$ and $d_{j}$ in $AM_{pd}$ and obtain an update protein–domain adjacency matrix $UAM_{pd}$ *.*

**Step 2:** Applying the domain-based collaborative filtering algorithm

Similarly, we can also obtain an original adjacency matrix $AM_{dp}$ and a co-occurrence matrix $CM_{dd}$ . Obviously, as for the matrix $AM_{dp}$ , there is $AM_{dp}=AM_{pd}^{T}$ *.* However, as for the matrix $CM_{dd}$ , for any two given domains $d_{i}$ and $d_{j}$ , there is $\;CM_{dd}\left(d_{i},d_{j}\right)=1$ , if and only if there is at least one common protein node existing between them; otherwise, there is $\;CM_{dd}\left(d_{i},d_{j}\right)=0$ . After normalizing $CM_{dd}$ , we can calculate the similarity between $d_{i}$ and $d_{j}$ as follows:

$$\mathrm{SMDD}\left(d_{i}\;,\;d_{j}\right)=\{\begin{array}{cc} \frac{\left|N\left(d_{i}\right)\cap N\left(d_{j}\right)\right|}{\sqrt{\left|N\left(d_{i}\right)\right|\times \left|N\left(d_{j}\right)\right|}}, & if\;k\neq r\\ 0, & \mathrm{Otherwise} \end{array},$$ (4)

where $\left|N\left(d_{i}\right)\right|$ represents the number of known proteins associated with $d_{i}$ in PDI, and $\left|N\left(d_{i}\right)\cap N\left(d_{j}\right)\right|$ represents the number of known proteins related to $d_{i}$ and $d_{j}$ simultaneously.

We can as well define the recommended standard and recommendation matrix as follows:

$$\mathrm{RMDP}=\mathrm{SMDD}\times AM_{dp}$$ (5)

$${Std}_{dk}p_{j}=\frac{1}{M}\times \overset{M}{\underset{i=1}{\sum }}\mathrm{RMDP}\left(d_{i},p_{j}\right)$$ (6)

Here, M means the number of domains in PDI . In particular, if there exists a domain node $d_{k}$ in the $i^{th}$ column of  RMDP satisfying $RMDP\left(d_{k},p_{j}\right)>Std_{d_{k\;}p_{j}}$ , then we further recommend the protein $d_{k}$ to domain $p_{\text{j}}$ . Thereafter, we also add a new association edge between $d_{k}$ and $p_{j}$ in $AM_{dp}$ and obtain an update association $UAM_{dp}$ .

**Step 3:** Mutual recommendation between proteins and domains

Based on the updated matrix $\;UAM_{pd}$ and $UAM_{dp}$ , the $\;UAM_{pd}$ is N×M dimension matrix, and $UAM_{dp}$ is M×N matrix. By transposing the matrix $AM_{dp}$ , it is obvious that we can construct the mutual recommendation matrix MRM as follows:

$$\mathrm{MRM}\left(p_{i}\;,\;d_{j}\right)=\{\begin{array}{cc} UAM_{pd}\left(p_{i}\;,\;d_{j}\right)+\;UAM_{dp}^{T}\left(p_{i}\;,\;d_{j}\right), & \mathrm{otherwise}\\ 1, & if\;\;\mathrm{UA}M_{pd}\left(p_{i}\;,\;d_{j}\right)=1\;and\;\;\mathrm{UA}M_{dp}^{T}\left(p_{i}\;,\;d_{j}\right)=1 \end{array}$$ (7)

For instance, according to Figure 1 and the given matrix $\;AM_{pd}=\left[\begin{array}{cc} \begin{array}{cc} 1 & 1\\ 1 & 0 \end{array} & \begin{array}{cc} 0 & 0\\ 1 & 0 \end{array}\\ \begin{array}{cc} 0 & 1\\ \begin{array}{c} 0\\ 0 \end{array} & \begin{array}{c} 1\\ 0 \end{array} \end{array} & \begin{array}{cc} 0 & 0\\ \begin{array}{c} 0\\ 0 \end{array} & \begin{array}{c} 1\\ 1 \end{array} \end{array} \end{array}\right]$ , we can obtain its corresponding matrices $CM_{PP}$ , SMPP , and  RMPD as follows:

$$\begin{array}{l} CM_{\mathrm{PP}}=\left[\begin{array}{ccccc} 0 & 1 & 1 & 1 & 0\\ 1 & 0 & 0 & 0 & 0\\ 1 & 0 & 0 & 1 & 0\\ 1 & 0 & 1 & 0 & 1\\ 0 & 0 & 0 & 1 & 0 \end{array}\right],\mathrm{SMPP=}\left[\begin{array}{ccccc} 0 & 0\mathrm{.5} & 0\mathrm{.71} & 0\mathrm{.5} & 0\\ 0\mathrm{.5} & 0 & 0 & 0 & 0\\ 0\mathrm{.71} & 0 & 0 & 1 & 0\\ 0\mathrm{.5} & 0 & 0\mathrm{.71} & 0 & 0\mathrm{.71}\\ 0 & 0 & 0 & 0\mathrm{.71} & 0 \end{array}\right],\\ \mathrm{RMPD=}\left[\begin{array}{cccc} 0.5 & 1.21 & 0.5 & 0.5\\ 0.5 & 0.5 & 0 & 0\\ 0.71 & 1.41 & 0 & 0.71\\ 0.5 & 1.21 & 0 & 0.71\\ 0 & 0.71 & 0 & 0.71 \end{array}\right] \end{array}$$

**FIGURE 2:**
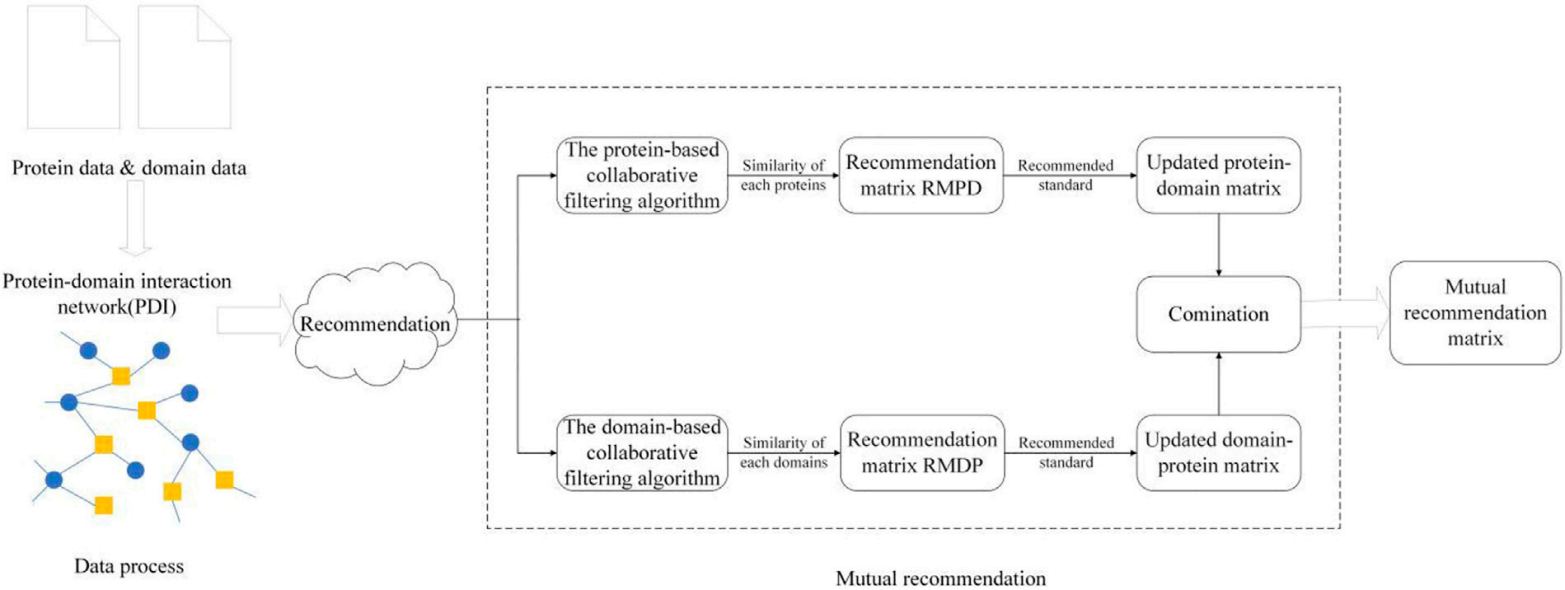
Flowchart of mutual recommendation.

To be specific, as illustrated in Figure 1, if tanking the domain node $d_{1}$ as an instance, then it is obvious that there are two protein nodes $p_{1}$ and $p_{2}$ associated with ${\text{d}}_{1}$ from the matrix $AM_{pd}$ . In addition, according to Eq. 2, we can as well obtain the recommended standard $RMPD\;\left(p_{3},d_{1}\right)=0.71\;>Std_{p3\;d1}=0.44$ . Hence, we will recommend the protein node $p_{3}$ to $d_{1}$ . In the same way, the protein node $p_{4}\;$ will be recommended to $d_{1}$ as well. On the contrary, $RMPD\;\left(p_{2},d_{2}\right)=0.5$ and $RMPD\;\left(p_{5},d_{2}\right)=0.5$ are less than the recommended standard $\;Std_{p2\;d2}$ = $Std_{p5\;d2}$ *=* 1.01. So there is no need to recommend the protein node $p_{2\;}$ and $p_{5}$ to $d_{2}$ . In addition, according to a previous description, it is obvious that these novel edges between $p_{3}$ and $d_{1}$ *,* $p_{4}$ and $d_{1}$ , $p_{1}$ and $d_{3}$ , $p_{3}$ and $d_{4}$ will be added to the original protein–domain association matrix $AM_{pd}$ in the same time. Similarly, we can apply the domain-based collaborative filtering algorithm. Thereafter, we can obtain a recommendation protein–domain adjacency matrix based on PDI. Finally, as shown in Figure 2. We can get the mutual recommendation matrix MRM.

### Construction of the Weighted Protein–Protein Interaction Network

For any two given protein $p_{i}$ and $p_{j}$ , we estimate the relationship between $p_{i}$ and $p_{j}$ by applying the Gaussian kernel interaction profile (van Laarhoven et al., 2011) and further obtain an N×N dimensional weight matrix between proteins WBP based on the mutual recommendation matrix MRM . $\;WBP\left(p_{i}\;,\;p_{j}\right)\;$ represents the relationship between protein $p_{i}$ and $p_{j}$ , and it can be defined as follows:

$$WBP\left(p_{i},\;p_{j}\right)=exp{\left(-\delta_{p}\|IP\left(d_{i}\right)-IP\left(d_{j}\right)\|\right)}^{2}$$ (8)

where

$$\delta_{p}=\frac{\delta_{p}^{\prime }}{\frac{1}{N}\sum_{i=1}^{N}\|IP{\left(d_{i}\right)\|}^{2}}$$ (9)

Here, $IP\left(d_{i}\right)$ and $IP\left(d_{j}\right)$ represents the vector at the $i^{th}$ and $j^{th}$ column of the mutual recommendation matrix MRM separately. $\delta_{p}$ is an adjustment coefficient, which controls kernel bandwidth based on normalizing the new bandwidth parameter $\;\delta_{p}^{\prime }$ .

### Calculate the Score of Multiple Features of Protein

Previous research has indicated that with similar functions, co-expressed and complex topologies are more likely to be essential proteins. Inspired by them, in this paper, we combine biological and topological features to detect potential proteins by subcellular localizations, gene expression data, and orthologous information and PPI networks.

**FIGURE 3:**
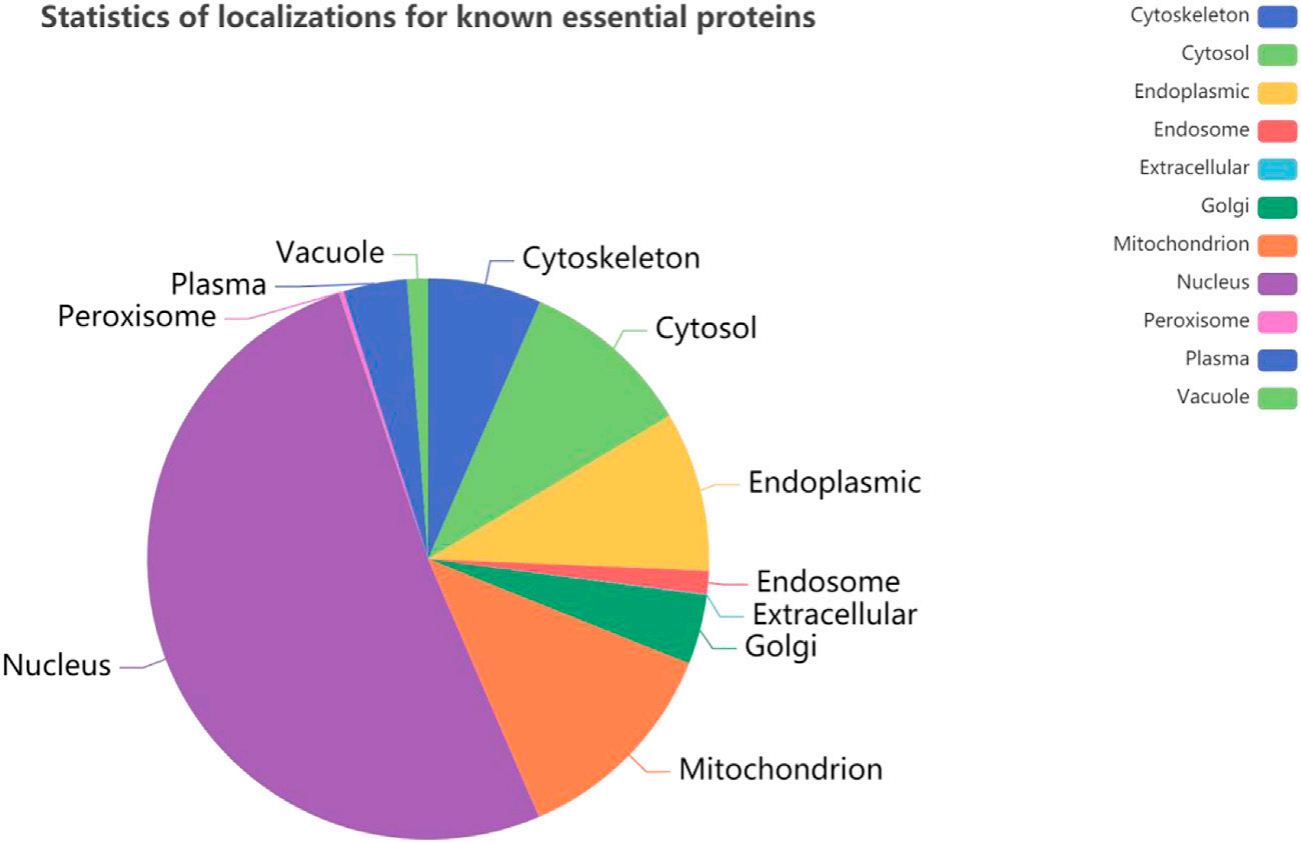
Statics of localization for known key proteins.

**FIGURE 4:**
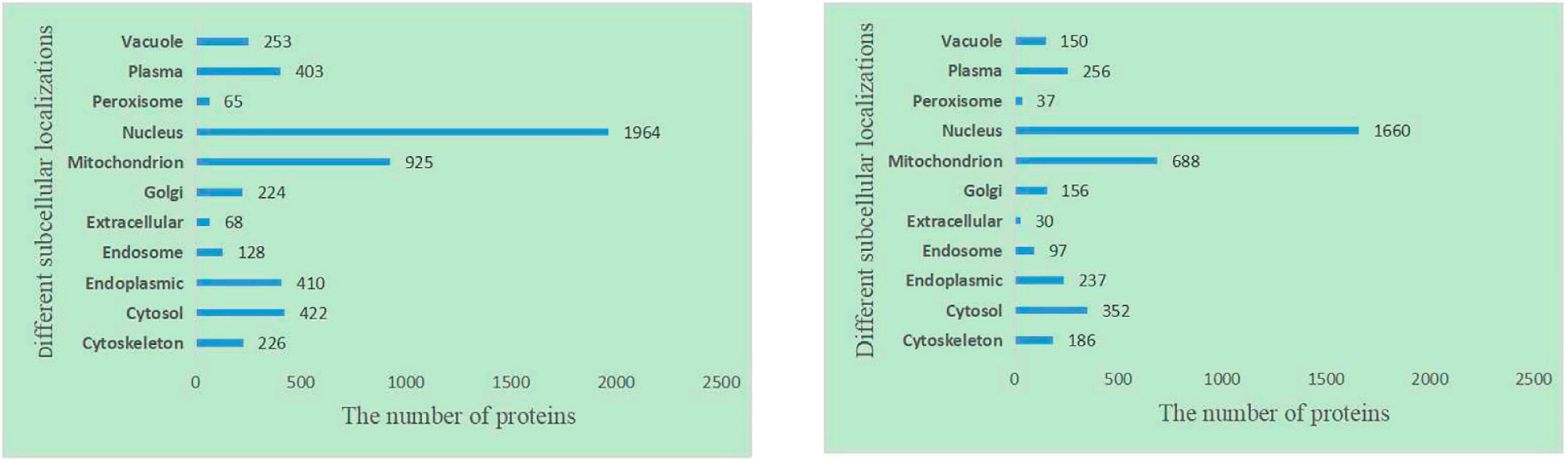
The number of proteins in each subcellular locations based on the DIP and Krogan protein databases.

It is obvious that the location information of a protein in a cell is an important characteristic of essential proteins. First, we analyze the 11 kinds of subcellular location relationship between the known essential proteins, and the Figure 3 statistical distribution of each subcellular location is shown in Figure 4. We can find that essential proteins are not randomly distributed in different subcellular locations, and essential proteins appear more often in the nucleus and mitochondrion, which means that proteins in the nucleus and mitochondrion are more possible to be essential proteins. What is more, from Figure 4, there are more essential proteins in the nucleus and mitochondrion and a few essential proteins in the peroxisome and extracellular, which provides us with convenience.

In order to distinguish the importance of different subcellular locations, let $N_{sub}$ means the number of all subcellular localizations and $\;N_{sub}\left(i\right)$ represent the number of proteins associated with the $i^{th}$ subcellular localization. Then $Ave_{sub}$ denotes the average number of proteins related to each subcellular localization. The score of the $i^{th}$ subcellular localization $Eve_{sub}\left(i\right)$ can be expressed as follows:

$$Ave_{sub}=\frac{\sum_{i=1}^{N_{sub}}N_{sub}\left(i\right)}{N_{sub}}$$ (10)

$$Eve_{sub}\left(i\right)=\frac{N_{sub}\left(i\right)}{Ave_{sub}}$$ (11)

Let $Sub_{\left(p_{k}\right)}$ represent the set of subcellular localizations associated with the protein $p_{k}$ . Therefore, for a given protein $p_{k}$ , its subcellular localization score $Pro_{sub}\left(p_{k}\right)$ is computed as the sum of the scores of all subcellular locations where it appears.

$$Pro_{Sub}\left(p_{k}\right)=\underset{i\varepsilon Sub_{\left(p_{k}\right)}}{\sum }Eve_{sub}\left(i\right)$$ (12)

Similar to describing subcellular scores, for any given protein $\;p_{k}$ , let $\;Pro_{ort}\left(p_{k}\right)$ mean the score of orthologous information. Hence, we can define its feature of orthology information score for $p_{k}$ as follows:

$$Pro_{Ort}\left(p_{k}\right)=\frac{Ort\left(p_{k}\right)}{max_{p_{i}\varepsilon PPI}\left\{Ort\left(p_{i}\right)\right\}}$$ (13)

We use the Pearson correlation coefficient (Priness et al., 2007) as a similarity measure of gene expression profiles to calculate the expression intensity of two genes.

$$PCC\left(p_{k},p_{r}\right)=\frac{1}{n-1}\overset{n}{\underset{i=1}{\sum }}\left(\frac{Exp\left(p_{k},i\right)-\bar{Exp\left(p_{k}\right)}}{\sigma \left(p_{k}\right)}\right)\left(\frac{Exp\left(p_{r},i\right)-\bar{Exp\left(p_{r}\right)}}{\sigma \left(p_{r}\right)}\right)$$ (14)

Here $Exp\left(p_{k},i\right)$ represents the expression level of $p_{k}$ at the $i^{th}$ time node. $\bar{Exp\left(\;p_{k}\right)}$ is the average gene expression value of protein $\;p_{k}$ , and $\sigma \left(p_{k}\right)$ is the standard deviation of protein $\;p_{k}$ . Thereafter, let $\;NG\left(p_{k}\right)$ denote the set of neighbors of protein $\;p_{k}$ . So we can compute its new functional score of protein $p_{k}$ as follows:

$$Pro_{Exp}\left(p_{k}\right)=\frac{exp\left(p_{k}\right)}{max_{p_{i}\varepsilon PPI}\left\{exp\left(p_{i}\right)\right\}}$$ (15)

where

$$exp\left(p_{k}\right)=\underset{p_{r}\varepsilon NG\left(p_{k}\right)}{\sum }PCC\left(p_{k},p_{r}\right)$$ (16)

It is a fact that essential proteins are more likely products of complex functions (Dezso et al., 2003). In addition, it is obvious that triangles have stable characteristics. Inspired by this, we further utilize the major triangle topological feature calculated by the original PPI network for obtaining each protein topological feature score. Therefore, for a given protein $p_{k}$ , we can calculate the topological feature score as follows:

$$Pro_{Tri}\left(p_{k}\right)=\frac{\sum_{p_{r}\varepsilon NG\left(p_{k}\right)}NG\left(p_{k}\right)\cap NG\left(p_{r}\right)}{NG\left(p_{k}\right)}$$ (17)

Based on the above formulas for any given protein $p_{k}$ , we can obtain the main topological and biological feature scores.

In order to effectively solve the problem of multifeature integration, we apply an improved entropy weight method (Dastbaz et al., 2018) to automatically generate the best parameters to integrate biological features. Based on the protein characteristics we have normalized, let $\left\{BF_{i1},BF_{i2},...BF_{iM}\right\}$ represent all features; then we can further construct an N×M dimensional matrix BF and an M×1 dimensional matrix PM as follows:

$$BF=\left[\begin{array}{ccc} BF_{11} & \cdots & BF_{1M}\\ \vdots & \ddots & \vdots \\ BF_{N1} & \cdots & BF_{NM} \end{array}\right]$$ (18)

$$PM=\left[\begin{array}{c} p_{1}\\ \vdots \\ p_{M} \end{array}\right]$$ (19)

Next, based on our normalized biological features, we can obtain the entropy value of each feature separately as follows:

$$e_{i}=-\frac{1}{lnN}\overset{N}{\underset{i=1}{\sum }}BF_{ij}.ln\left(BF_{ij}\right)$$ (20)

Therefore, for the $i^{th}$ protein biological feature, we can calculate the entropy weight of each feature by the following formula:

$$w_{j}=\frac{\left(1-e_{i}\right)}{\overset{M}{\underset{i=1}{\sum }}\left(1-e_{i}\right)}$$ (21)

Based on the above formula, for a given protein $p_{k}$ , we can further calculate its integrated biological score as follows:

$$pro_{Bio}\left(p_{k}\right)=\overset{M}{\underset{k=1}{\sum }}w_{j}BF_{kj}$$ (22)

Finally, according to the above Eq. 18, for any given protein ${\text{p}}_{\text{k}}$ , we can further obtain its initial score as follows.

$$pro_{score}\left(p_{k}\right)=\text{λ}\times pro_{Bio}\left(p_{k}\right)+\left(1-\text{λ}\right)\times Pro_{Tri}\left(p_{k}\right)$$ (23)

Here, λ is a proportion parameter with a value between 0 and 1.

### Construction of the Prediction Model Collaborative Filtering Model-Based Method

According to  WBP , our prediction model CFMM can apply improved PageRank to identify potential proteins. Let $WP\left(p_{k}\;,\;p_{r}\right)=\frac{WBP\left(p_{k}\;,\;p_{r}\right)}{{\left(1+\text{max}\left(WBP\left(p_{k}\;,\;p_{r}\right)\right)\right)}^{2}}$ , and for any two given proteins $p_{k}$ and $p_{r}$ , we can define the distribution rate possibility matrix as follows:

$$DRPM_{\left(p_{k}\;,\;p_{r}\right)}=\{\begin{array}{cc} WP\left(p_{k}\;,\;p_{r}\right)\times \frac{pro_{score}\left(p_{r}\right)}{\sum_{p_{i}\varepsilon NG\left(p_{k}\right)}pro_{score}\left(p_{i}\right)} & if\;WP\left(p_{k}\;,\;p_{r}\right)\neq 0\\ 0, & Otherwise \end{array}$$ (24)

Based on the above distribution rate matrix *DRPM*, let a possibility vector $pro_{Score}\left(t\right)$ , $pro_{Score}\left(t+1\right)$ mean the score vector of protein at the $t^{th}$ and $t+1^{th}$ time separately; therefore, we can iteratively compute the protein ranks as follows:

$$pro_{Score}\left(t+1\right)=\alpha \times pro_{Score}\left(t\right)\times DRPM+\left(1-\alpha \right)\times pro_{Score}\left(0\right)$$ (25)

Here the parameter α ∈ (0, 1) in order to adjust the proportion $pro_{score}\left(t\right)$ and initial score $\;pro_{score}\left(0\right)$ .

Based on the above descriptions, our prediction method CFMM can be concisely described as follows.

**Table:** 

Algorithm CFMM
Input: original protein–domain network, original PPI network subcellular data, orthologous data, expression data, the iteration termination condition ε , and adjustment parameter α .
Output: the final score of proteins.
Step 1: Apply the protein-based collaborative filtering algorithm by Eqs 1–3.
Step 2: Apply the domain-based collaborative filtering algorithm by Eqs 4–6.
Step 3: Calculate the weights between proteins based on the MRM based on Eqs 7–9.
Step 4: Compute the protein feature score based on Eqs 10–23.
Step 5: Establishing distribution network based on Eq. 24.
Step 6: Let t=t+1 , calculate $pro_{score}\left(t+1\right)$ according to Eq 26.
Step 7: Repeat step6 until $pro_{score}\left(t+1\right)-pro_{score}{\left(t\right)}_{2}<\varepsilon$ .
Step 8: Sorting the proteins scores $pro_{score}\left(t+1\right)$ through descending order.

## Performance Evaluation

### Comparison Between Collaborative Filtering Model-Based Method and 14 Representative Methods

**FIGURE 5:**
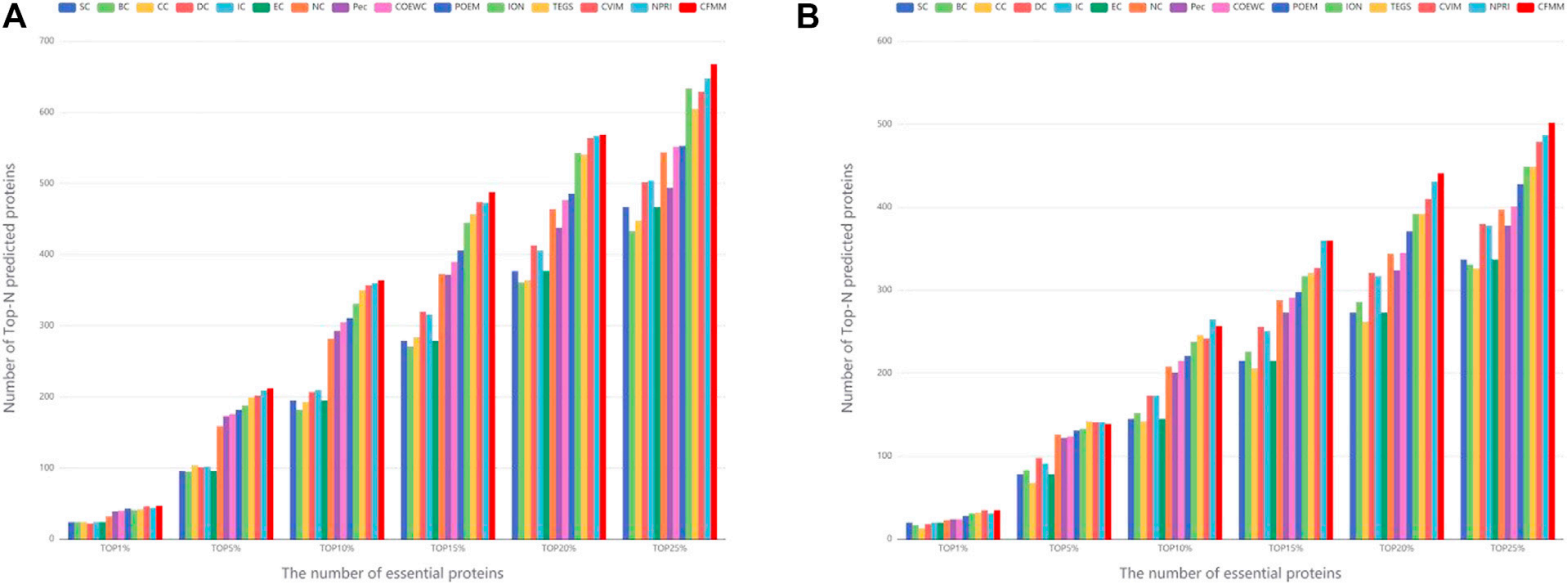
**(A)** Performances achieved by CFMM and other candidate methods under the DIP database. **(B)** Performances achieved by CFMM and other candidate methods under the Krogan database.

In order to further evaluate the performance of CFMM in this section, two different datasets, the DIP database and the Krogan database, are adopted to compare CFMM with 14 competitive detection models, which include DC (Hahn and Kern, 2005), SC (Estrada and Rodríguez-Velázquez, 2005), BC (Joy et al., 2005), EC (Bonacich, 1987), IC (Stephenson and Zelen, 1989), CC (Wuchty and Stadler, 2003), NC (J. Wang et al., 2012), ION (Peng et al., 2012), Pec (Li et al., 2012), CoEWC (Zhang et al., 2013), POEM ((Zhao et al., 2014), TEGS (Zhang W. et al., 2018), CVIM (S. Li et al., 2020), and NPRI (Z. Chen et al., 2020). For the purpose of observing the accuracy of the experiment more intuitively, we chose to use a bar graph to compare the 1, 5, 10, 15, 20, and top 25% of each method. Figure 5 shows that the comparison of the identifying results of different algorithms on the DIP and Krogan database separately. From Figure 5A, the newly put forward CFMM method detected a larger number of essential proteins in the top 1–25% compared with 14 other competitive methods. It is obvious that CFMM can reach the accuracy of 92.16, 83.14, 71.37, 63.87, 55.84, and 52.43% in the top 1, 5, 10, 15, 20, and 25% predicted candidate key proteins based on the DIP database. Among the top 25% proteins predicted by the CFMM method, there are 668 proteins correctly detected, which indicates that the CFMM method has superior advantages over other methods. From Figure 5B, we can see that CFMM can reach the accuracy of 94.59, 75.54, 70.03, 65.34, 60.08, and 54.68% in the top 1, 5, 10, 15, 20, and 25%, which are superior to all 14 advanced methods, except that in the top 10% CFMM-predicted 257 proteins, they are a little lower than NPRI. Therefore, we can make a conclusion that CFMM always obtains the better prediction accuracy from the top 1% to the top 25%.

### Validated by Jackknife Methodology

**FIGURE 6:**
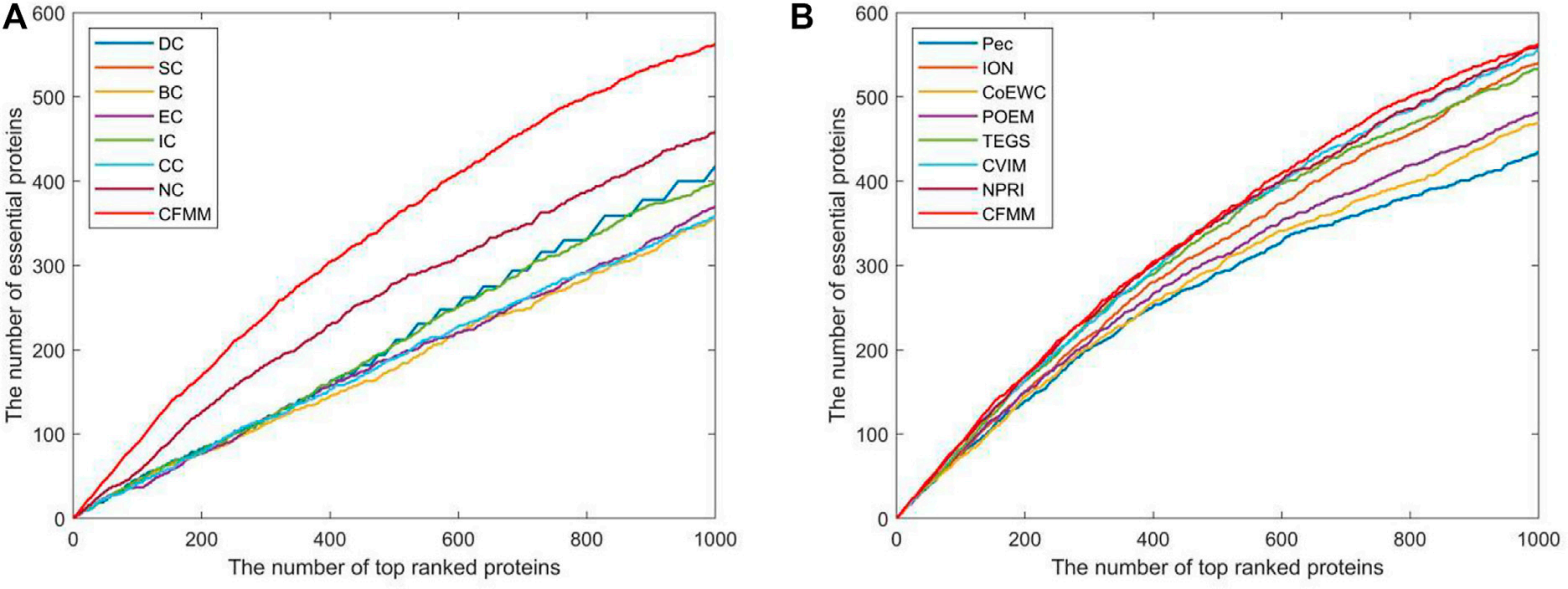
Comparison of jackknife curves of CFMM and 14 other methods under the DIP database. **(A)** Comparison between CFMM and DC, IC, EC, SC, BC, CC, and NC. **(B)** Comparison between CFMM and Pec, CoEWC, POEM, ION, TEGS, CVIM, and NPRI.

**FIGURE 7:**
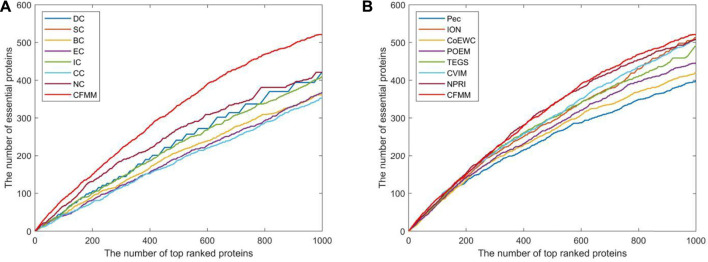
Comparison of jackknife curves of CFMM and 14 other methods under the Krogan database. **(A)** Comparison between CFMM and DC, IC, EC, SC, BC, CC, and NC. **(B)** Comparison between CFMM and Pec, CoEWC, POEM, ION, TEGS, CVIM, and NPRI.

Due to the jackknife methodology (Holman et al., 2009) that can evaluate the advantages and disadvantages of the prediction model, in this section, we will apply the jackknife method to assess the predictive effect of our proposed mode CFMM. Figures 6, 7 show the experimental comparisons between CFMM and 14 advanced competitive methods based on the first 1,000 candidate proteins. By observing Figure 6A, it is obvious that CFMM can achieve better performance than the seven network topology-based methods including DC, SC, BC, EC, IC, CC, and NC. What is more, Figure 6B shows that the performance of CFMM is better than the other seven methods that are based on the combination of biological information of proteins and PPI networks including Pec, CoEWC, POEM, ION, TEGS, CVIM, and NPRI. From Figure 7A, we can easily conclude that the CFMM is advanced than these centrality-based methods including DC, IC, EC, BC, CC, SC, and NC. Although the performance curves of CFFM and NPRI overlap partially, as the number of candidate proteins increases to 450, the predictive performance of CFMM will be significantly higher than that of NPRI. Therefore, based on the above description, we can make a conclusion that the performance of CFMM is not only superior to the first category of methods, such as DC, SC, BC, EC, IC, CC, and NC, but also better than these multiple biological data methods including Pec, CoEWC, POEM, ION, TEGS, CVIM, and NPRI.

### Differences Between Collaborative Filtering Model-Based Method and Competitive Methods

**FIGURE 8:**
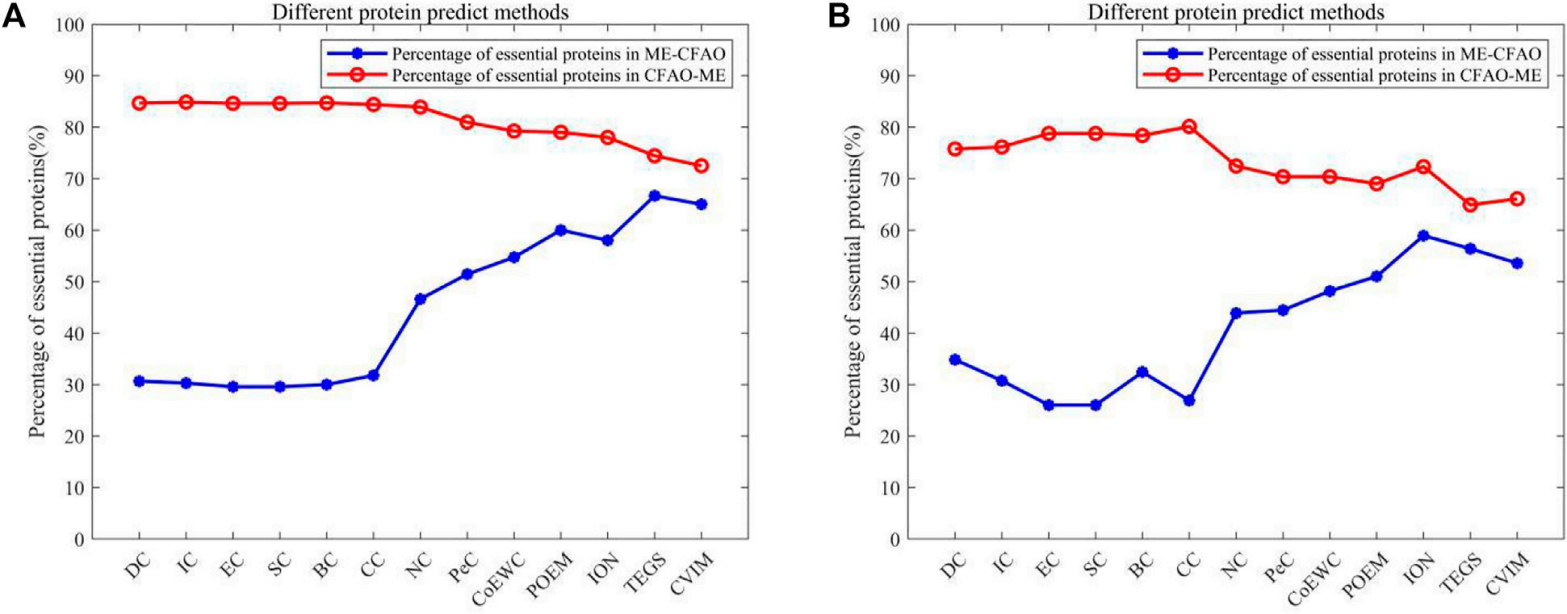
The *X*-axis represents different protein predicted methods. The *Y*-axis represents the proportion of essential proteins in {ME−CFMM} or {CFMM−ME}.

In order to further prove the accuracy of the CFMM model, we will analyze the differences between CFMM and other models based on the top 100 predicted proteins under the DIP database and the Krogan database separately, and comparison results are shown in Tables 2, 3, respectively. Here ME denotes one of the 14 competitive methods. |CFMM∩ME| represents the number of essential proteins predicted by both CFMM and ME. |CFMM−ME| denotes the number of essential proteins recognized by the CFMM but not by ME, and |ME−CFMM| means the number of key proteins predicted by ME but ignored by CFMM. In addition, {CFMM−ME} represents the set of key proteins recognized by CFMM but not by ME. {ME−CFMM} means the set of essential proteins predicted by ME but not by CFMM. Hence, Tables 2, 3 show the difference between the 14 competitive methods and CFMM under the DIP and Krogan datasets separately. Figure 8 indicates that CFMM can achieve much better predictive performance than all these competing methods as a whole.

**TABLE 2:** The connection and difference between CFMM and 14 competing methods based on the top 100 ranked proteins in the DIP database.

Different methods (ME)	|CFMM∩ME|	|CFMM−ME|	Percentage of key proteins in (%) {CFMM−ME}	Percentage of key proteins in (%) {ME−CFMM}
DC	6	94	88.30	42.55
IC	6	94	88.30	40.43
EC	6	94	88.30	32.98
SC	6	94	88.30	32.98
BC	5	95	88.42	41.05
CC	5	95	88.42	37.89
NC	35	65	89.23	36.92
Pec	46	54	87.04	59.26
CoEWC	47	53	84.91	54.72
POEM	56	44	84.09	65.91
ION	38	62	88.71	70.97
TEGS	58	42	80.95	64.29
CVIM	44	56	85.71	83.93
NPRI	76	24	91.67	87.50

**TABLE 3:** The connection and difference between CFMM and 14 competing methods based on the top 100 ranked proteins in the Krogan database.

Different methods (ME)	$\left|\text{CFMM}\cap^{\text{​}}\text{ME}\right|$	|CFMM−ME|	Percentage of key proteins in (%) {CFMM−ME}	Percentage of key proteins in (%) {ME−CFMM}
DC	17	83	84.34	42.17
IC	12	88	85.23	44.32
EC	5	95	86.32	38.95
SC	5	95	86.32	38.95
BC	8	92	85.87	40.22
CC	5	95	86.32	43.16
NC	48	52	88.46	50.00
Pec	43	57	77.19	56.14
CoEWC	41	59	77.97	52.54
POEM	45	55	85.45	58.18
ION	30	70	82.86	65.71
TEGS	58	42	80.95	52.38
CVIM	67	33	75.76	72.73
NPRI	61	39	76.92	53.85

### Validation by Receiver Operating Characteristic Curve

**FIGURE 9:**
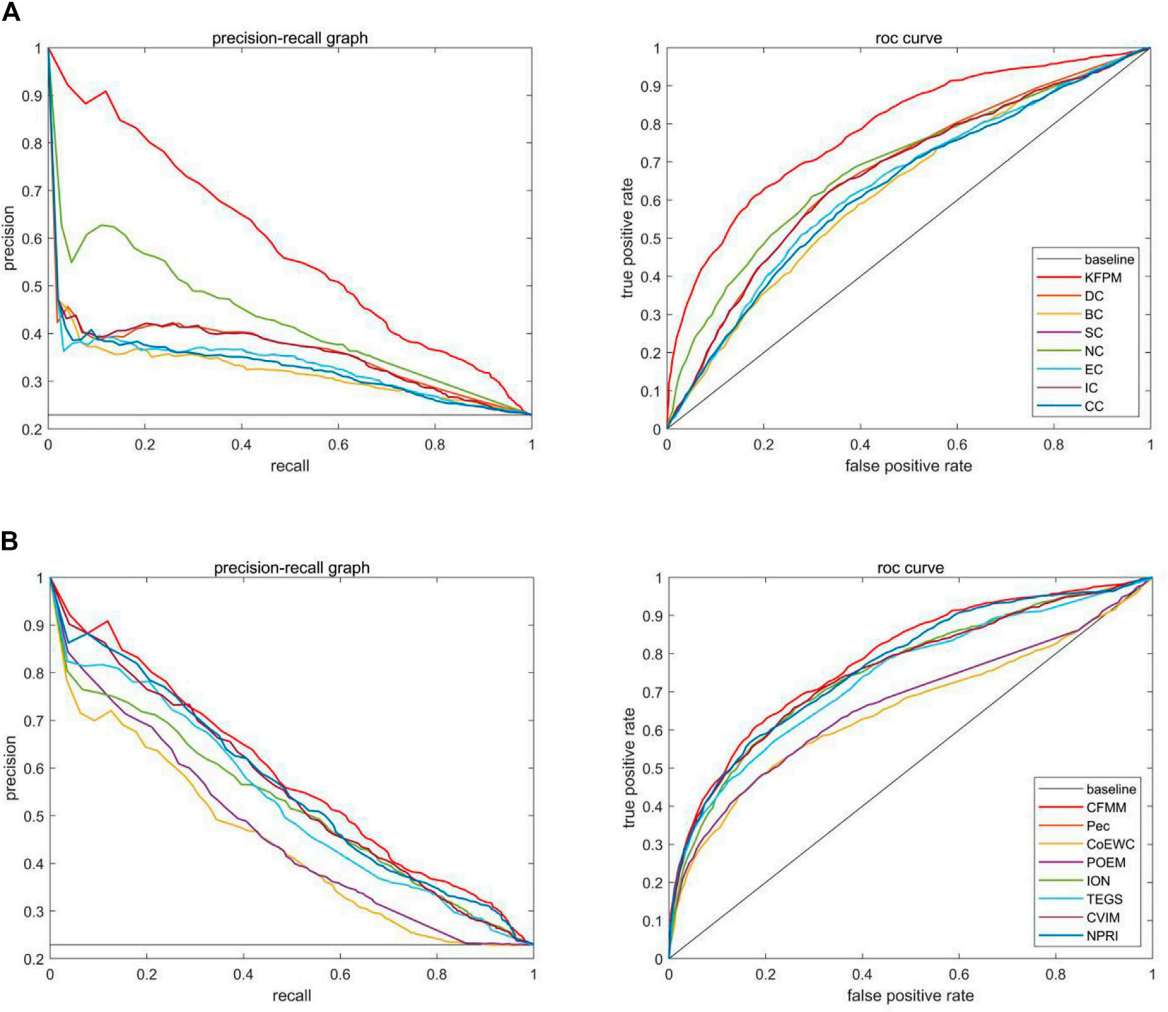
The precision recall (PR) curves and receiver operating characteristic (ROC) curves between CFMM and other advanced methods based on the DIP database. **(A)** The PR curves and the ROC curves of DC, BC, SC, NC, EC, IC, and CC. **(B)** The PR curves and the ROC curves of Pec, CoEWC, POEM, ION, TEGS, CVIM, and NPRI.

**FIGURE 10:**
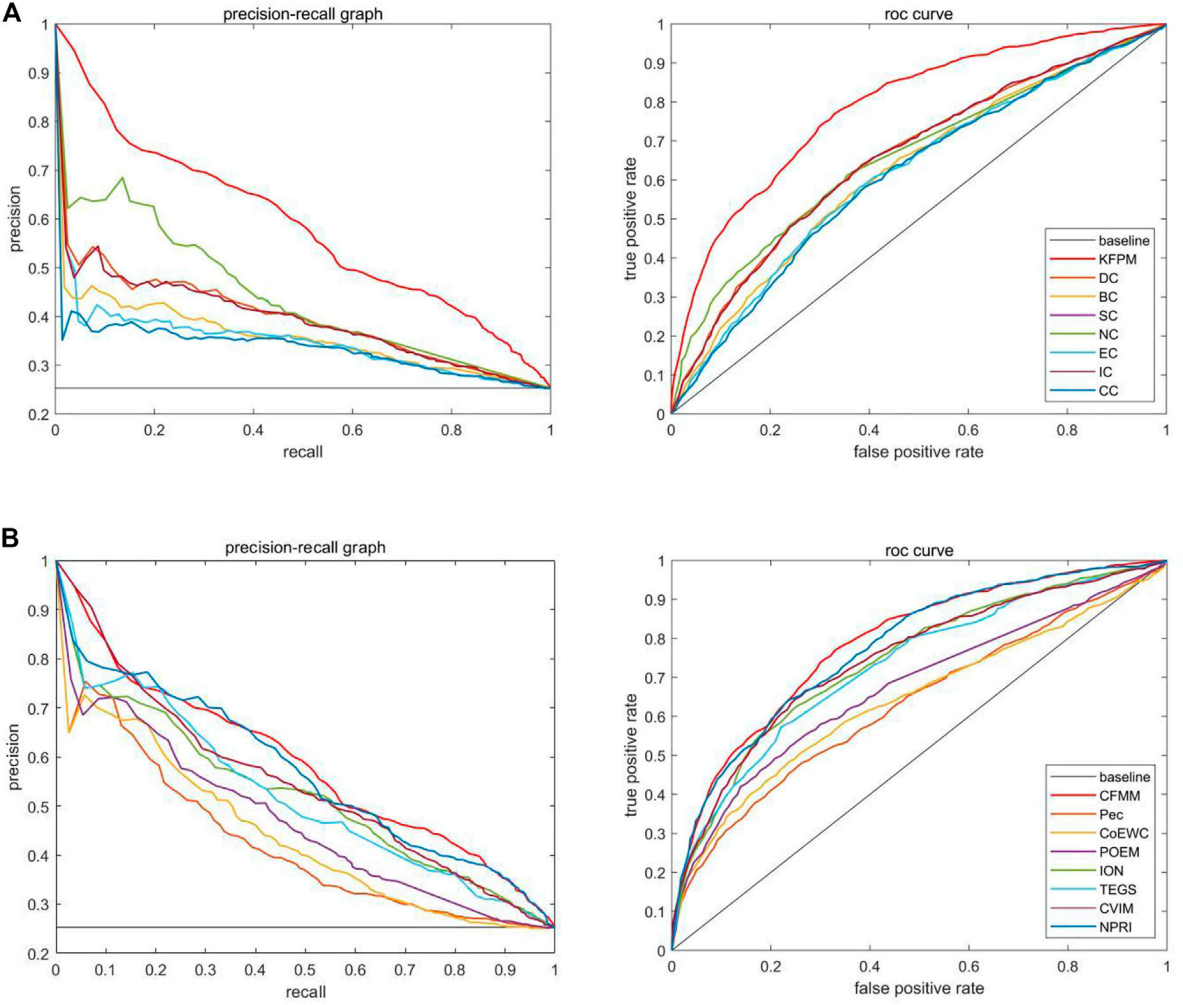
The PR curves and ROC curves between CFMM and other advanced methods based on the Krogan database. **(A)** The PR curves and the ROC curves of DC, BC, SC, NC, EC, IC, and CC. **(B)** The PR curves and the ROC curves of Pec, CoEWC, POEM, ION, TEGS, CVIM, and NPRI.

The receiver operating characteristic (ROC) curve and precision recall curve (PR) are used to scientifically prove the performance of the prediction model. The area under the curve (AUC) is used to evaluate the performance of the prediction method. The closer the AUC value is to 1, the better the prediction performance of the method. The curve can be plotted by the ratio of true positive rate (TPR) to false positive rate (FPR) according to different thresholds (Peng et al., 2020). Hence, we will further utilize the ROC curves to compare CFMM with other advanced models. Figures 9, 10 indicate that the ROC curves and PR curves of CFMM and other competitive models are based on the DIP and Krogan databases separately. It is obvious that CFMM has a higher AUC curve than other competitive models. Although we can see that the ROC curve of CFMM and the NPRI ROC curves overlap slightly, the AUC value of CFMM is higher than NPRI. Finally, in order to prove the applicability of CFMM, we will further test it in the Gavin database and compare with other methods. The experimental results are shown in Tables 4, 5.

**TABLE 4:** The area under the curve (AUC) value of each method under the DIP and Krogan databases.

Method	AUC (DIP)	AUC (Krogan)
CFMM	0.7854	0.7877
NPRI	0.7683	0.7768
CVIM	0.7559	0.7458
TEGS	0.7386	0.7287
ION	0.7522	0.7413
POEM	0.6662	0.6726
CoEWC	0.6513	0.6404
Pec	0.6329	0.6316
CC	0.6291	0.6114
IC	0.6657	0.6573
EC	0.6384	0.6167
NC	0.6879	0.6584
SC	0.6384	0.6167
BC	0.625	0.6248
DC	0.6704	0.6583

**TABLE 5:** The number of key proteins recognized by CFMM and other methods based on the Gavin database.

Methods	Top 1% (19)	Top 5% (93)	Top 10% (196)	Top 15% (279)	Top 20% (371)	Top 25% (464)
DC	7	36	101	158	222	264
IC	16	55	119	163	213	254
CC	11	45	93	135	180	221
BC	9	40	85	122	162	201
SC	0	17	87	130	190	240
EC	0	38	94	134	166	209
NC	11	51	123	170	213	259
CoEWC	16	69	136	190	237	275
Pec	15	69	142	193	238	285
ION	17	73	150	207	263	312
POEM	17	74	148	199	249	296
CVIM	16	80	160	219	271	322
NPRI	16	75	153	221	278	323
CFMM	19	84	162	222	280	332

### The Analysis of Parameter

In this section, we discuss the effect of the two self-defined parameters α and λ on the prediction results of CFMM. We set the parameter α to vary from 0.1 to 0.9, then the CFMM algorithm is ran nine times from α = 0.1 to α = 0.9 separately. Finally, the number of true essential proteins identified by CFMM based on the DIP and Krogan databases are shown in Tables 6, 7 separately. Here we select from the top 1% to the top 25% of the proteins identified by CFMM. The prediction accuracy is based on the number of essential proteins that are truly identified. It is obvious that the closer α value is to 1, the higher the prediction accuracy CFMM can achieve. So, we consider that the parameter α on all the databases is 0.9, which can achieve the best performance. When α is set to 0.9, and λ is set to 0.65, the amount of true essential protein is closest to its average level. Therefore, as a result, we will set α and λ on the DIP and Krogan databases to 0.9 and 0.65 separately, while for the Gavin database, the optimum parameters α and λ will be set to 0.9 and 0.8, respectively.

**TABLE 6:** Effects of the parameter α to CFMM based on the DIP database.

α	0.1	0.2	0.3	0.4	0.5	0.6	0.7	0.8	0.9
Rank									
Top 1% (51)	47	47	47	47	47	47	47	47	47
Top 5% (255)	206	208	207	208	209	209	210	213	212
Top 10% (510)	357	357	358	361	361	359	358	360	364
Top 15% (764)	469	473	474	476	480	483	485	485	488
Top 20% (1,019)	572	574	573	573	571	575	576	573	569
Top 25% (1,274)	650	653	657	656	658	661	665	667	668

**TABLE 7:** Effects of the parameter α to CFMM based on the Krogan database.

α	0.1	0.2	0.3	0.4	0.5	0.6	0.7	0.8	0.9
Rank									
Top 1% (51)	36	36	36	36	36	36	35	35	35
Top 5% (255)	141	140	140	139	140	140	140	138	139
Top 10% (510)	255	255	253	254	256	254	256	256	257
Top 15% (764)	369	366	364	365	365	363	360	360	360
Top 20% (1,019)	442	443	442	444	444	443	441	441	441
Top 25% (1,274)	497	496	497	496	498	499	499	501	502

## Discussion

Accumulating evidence have shown that prediction of essential proteins is important for the development of an organism in biological process, complex disease diagnoses, and drug design. However, the requirement of identifying key protein prediction accuracy is not satisfied only through biological experiments and relying on the topological characteristics of the PPI network. In this manuscript, we constructed an original protein–domain network by combining protein and domain associations first. Then we formulated the prediction of potential essential proteins as a problem of the recommendation system and obtained an updated recommendation network through applying a novel mutual recommendation between protein and domain to the original association network. Next, after we integrate the biological features, we combine with the major topological features to obtain the initial protein score. Finally, we design a novel distribution rate matrix and apply an iterative algorithm based on the improved PageRank algorithm to calculate protein scores iteratively. In addition, we apply the CFMM method on the DIP database, Krogan database, and Gavin database to testify the performance, respectively. Experiments show that CFMM can achieve better performance than other advanced methods. In future work, we will use multi-information fusion method to integrate various information related to proteins and machine learning methods to further improve the prediction performance (Peng et al., 2017; Zhou et al., 2019).

## Data Availability

The datasets presented in this study can be found in online repositories. The names of the repository/repositories and accession number(s) can be found in the article/Supplementary Material.
